# Concerning Dr. Gorgas' "Series of Questions for Dental Students."

**Published:** 1886-02

**Authors:** 


CONCERNING DR. GORGAS' "SERIES OF QUESTIONS FOR DENTAL STUDENTS."—*From Dental Cosmos.*—"The object of this work is to facilitate the study of dental science and its collaterals and comprises leading questions on all the branches belonging to the course of study pursued by the dental student, —embracing dental histology, dental pathology, dental surgery, dental prosthesis, dental materia medica and therapeutics, general anatomy, physiology, chemistry,—organic and inorganic, —and metallurgy. The questions in these different departments seem to have been in the main well chosen, and correct answers to them would indicate a very respectable familiarity with the several subjects. No student who finds himself able to answer the questions therein contained need doubt his ability to pass a satisfactory examination; and there are not many practitioners who would not be benefitted by the mental effort and by the research needed to enable them to give replies satisfactory to themselves to these interrogatories."

---

*From The Southern Dental Journal.*

"Prof. Gorgas' fertile brain is ever at work to give to dentistry a substantial aid in its standard literature. This work is similar to one issued several years ago on dental science, which proved very valuable to students, yet its field is much broader, covering the entire curriculum of the student. Sometimes it is easier to ask than to answer questions—at least the student thinks so, and to him this book is of great importahce, as he will be led through the whole course of instruction, and can easily find when he is deficient."

---

*From The Archives of Dentistry, Dr. C. W. Spalding, Editor.*

"This work is a valuable guide to the dental student, and points out what he should learn, hence will save a great deal of valuable time. The "Quiz" has become an indispensable part of the course in all colleges."

---

*From The Dental Practitioner.*

"This work, as the preface states: "comprises leading questions on all the branches belonging to the course of study pursued by the dental student and its object is to facilitate the study of Dental Science and its collateral branches." A careful examination of the book will show that if it fails to accomplish the object for which it has been prepared it will only be for the reason that it is not fully appreciated by the profession. It comes from the pen of a well-known author and fills a want long felt by many dentists. While of value to the student of any College of Dentistry, it will have increased value to the professional student in active practice. It is a splendid plan of study for the young and old practitioner. We are sure that if every dentist would faithfully work out the answers and their suggestive line of study limitedly, that in a short period greater unanimity of knowledge in answering the questions of patients would exist in all communities."

---

*From The Independent Practitioner.*

"For the dental student such a work is invaluable. It not only gives proper direction to his duties, but it teaches him conciseness and preciseness. In preparing for examination it becomes an essential. Nor is it alone the student who will find it of value. The practitioner, who would retain in his memory the teachings of his College days, and who would keep abreast the march of progress, would do well to take up a chapter occasionally, and ask of himself the questions which it contains. If he be not at times astonished that he should have forgotten so much, shocked at his own ignorance, and thereby spurred on to a renewed application to his text-books, his experience will be different from that of most men.

The many years which Prof. Gorgas has spent as a teacher in our dental colleges especially qualifies him for the preparation of such a work as this. Nor is it the training of experience alone that he brings to the task. He has the broad views, the general culture, and the discriminating mind that are so necessary to the successful teacher, and all these qualities are amply exhibited in this excellent series of questions, which covers almost the whole field of dental research."

---

Dr. Richard Grady, Secretary of the "Maryland State Board of Dental Examiners," writes as follows concerning this work: "The book cannot be recommended too highly. To those engaged as students or teachers of dentistry, it is almost indispensable, and to those who are not, but simply practitioners a perusal of it will be profitable. Your position as a teacher of many years experience has rendered you familiar with nearly all that is best worth knowing in Dental Science, and your extensive observation and well-earned reputation in practice makes you a competent judge as to what is best suited for the requirements of dental students; as would therefore be expected from one whose works already stand high in the estimation of the profession, your work is essentially a practical one. The first "Questions on Dental Science" were so favorably received that it is hardly necessary to speak for "Series of Questions for the Dental Student," the universal welcome of which they are so deserving."

---

Of the 11th edition of the "Principles and Practice of Dentistry," Dr. J. N. Farrar, of New York, writes as follows: "I have just been reading your new work, "Principles and Practice of Dentistry," &c., and I must say it is the best and most scholarly work that I have read from any American author on dentistry."

---

Dr. George Watt, editor of the *Ohio State Journal of Dental Science*, writes as follows concerning the eleventh edition of the "Princtples and Practice of Dentistry." "This splendid book is undoubtedly *the* work on modern dental practice and reflects great credit on its editor—we are tempted to say author, for it is virtually a new work by Prof. Gorgas." "We have carefully examined the thousand pages and find little or nothing in them to criticise." "Every reader of the *Journal* should own a copy for reference, if not for study."

